# Palliative Care Knowledge and Planning in U.S. Adults

**DOI:** 10.1093/geroni/igab046.2856

**Published:** 2021-12-17

**Authors:** Amy Albright, Rebecca Allen

**Affiliations:** 1 VA Maine HCS, Gorham, Maine, United States; 2 University of Alabama, Tuscaloosa, Alabama, United States

## Abstract

Palliative care knowledge and health literacy are frequently underestimated in American adults; for example, as measured by the Newest Vital Sign (Weiss et al., 2005), 79.2% (n = 247) of participants within a Geriatrics Clinic sample displayed “adequate” functional health literacy, while 11.8% (n = 37) scored within the “possibly limited” range, and 9.0% (n = 28) scored within the “highly limited” range. There was additionally a significant association between health literacy and age (r = .15, p < .01) within this sample. The Palliative Care Knowledge Scale (PaCKS; Kozlov et al., 2018) was administered to participants, and higher scores indicated a greater knowledge of palliative care. This construct is particularly important to measure, as racial/ethnic disparities exist within this domain; for example, African Americans may have lower overall knowledge of palliative care services and advance care planning than non-Hispanic Whites (Noh et al., 2018). In the current study, knowledge of palliative care was measured using the PaCKS (Kozlov et al., 2018), and scores represented the widest possible range of 0 to 13 (M = 7.68, SD = 4.08). There was a significant correlation between age and PaCKS score (r = .12, p < .05), as palliative care knowledge increased with age. Females scored significantly higher (M = 8.29, SD = 3.91) than males (M = 6.81, SD = 4.18), t(309) = 3.18, p < .001. There was no main effect of race on palliative care knowledge, and post-hoc analysis using Tukey HSD did not demonstrate significant differences between groups.

